# Ketogenic Diet Has Moderate Effects on the Fecal Microbiota of Wild-Type Mice

**DOI:** 10.3390/nu15214629

**Published:** 2023-10-31

**Authors:** Nadine Rohwer, Racha El Hage, Christopher Smyl, Soeren Ocvirk, Tobias Goris, Tilman Grune, Alexander Swidsinski, Karsten-H. Weylandt

**Affiliations:** 1 Medical Department B, Division of Hepatology, Gastroenterology, Oncology, Hematology, Endocrinology and Diabetes, Brandenburg Medical School, University Hospital Ruppin-Brandenburg, 16816 Neuruppin, Germany; nadine.rohwer@mhb-fontane.de; 2 Faculty of Health Sciences, Joint Faculty of the Brandenburg University of Technology Cottbus-Senftenberg, Brandenburg Medical School and University of Potsdam, 14476 Potsdam, Germany; 3 Department of Molecular Toxicology, German Institute of Human Nutrition Potsdam-Rehbruecke, 14558 Nuthetal, Germany; 4 Department of Vascular Surgery, University Hospital Ruppin-Brandenburg, Brandenburg Medical School, 16816 Neuruppin, Germany; racha.elhage@mhb-fontane.de; 5 Medical Department, Division of Hepatology and Gastroenterology, Campus Virchow-Klinikum, Charité Universitätsmedizin Berlin, 13353 Berlin, Germany; 6 Intestinal Microbiology Research Group, German Institute of Human Nutrition Potsdam-Rehbruecke, 14558 Nuthetal, Germany; 7 ZIEL—Institute for Food and Health, Technical University of Munich, 85354 Freising-Weihenstephan, Germany; 8 Medical Department, Division of Hepatology and Gastroenterology, Campus Mitte, Charité Universitätsmedizin, 10117 Berlin, Germany; 9 Department of General Hygiene, Institute of Public Health, M Sechenov First Moscow State Medical University (Sechenov University), 119991 Moscow, Russia

**Keywords:** gut, ketogenic diet, ketosis, microbiome

## Abstract

The ketogenic diet (KD) is a high-fat, low-carbohydrate diet that has been reported to have neuroprotective effects. The health effects of KD might be linked to an altered gut microbiome, which plays a major role in host health, leading to neuroprotective effects via the gut-brain axis. However, results from different studies, most often based on the 16S rRNA gene and metagenome sequencing, have been inconsistent. In this study, we assessed the effect of a 4-week KD compared to a western diet (WD) on the colonic microbiome of female C57Bl/6J mice by analyzing fecal samples using fluorescence in situ hybridization. Our results showed distinct changes in the total number of gut bacteria following the 4-week KD, in addition to changes in the composition of the microbiome. KD-fed mice showed higher absolute numbers of *Actinobacteria* (especially *Bifidobacteria* spp.) and lower absolute levels of *Proteobacteria*, often linked to gut inflammation, in comparison with WD-fed mice. Furthermore, an increased abundance of the typically rare genus *Atopobium* was observed. These changes may indicate the possible anti-inflammatory effects of the KD. However, since the overall changes in the microbiota seem low, the KD effects might be linked to the differential abundance of only a few key genera in mice.

## 1. Introduction

The ketogenic diet (KD) is characterized by high-fat and low-carbohydrate dietary intake, leading to a state of ketosis [1]. It has been mainly used as a therapeutic approach for pharmaco-resistant epilepsy in children [2], and it is increasingly considered for other neurological disorders [3]. Ingestion of KD forces the body to utilize fatty acids instead of glucose as its primary energy source, resulting in restricted glycolysis, increased fatty acid oxidation, and, ultimately, a significant increase in the generation of ketone bodies. As a consequence of the elevation of fat-derived ketones, a metabolic switch can occur, in which the body obtains energy from the metabolism of ketone bodies. In addition to these metabolic alterations, KD has also been shown to modulate the levels of hormones, neurotransmitters, and neuropeptides and to affect key signaling pathways, such as PPARs, AMP-activated kinase, and mammalian target of rapamycin [1,4,5]. Furthermore, several studies have indicated that KD has anti-inflammatory effects and profoundly affects mitochondria by stimulating mitochondrial biogenesis, improving mitochondrial function, and decreasing oxidative stress [6]. The mechanisms implicated in the neuroprotective effects of KD are particularly those involving the aforementioned alterations in cellular energy metabolism and increased mitochondrial activity due to this diet [7]. Moreover, KD and ketone bodies are increasingly being studied for their therapeutic efficacy in different non-neurological disorders, including obesity, non-alcoholic fatty liver disease, heart failure, and cancer [7]. KD is applied in a variety of protocols that differ in caloric and macronutrient contents. For instance, the classical ketogenic diet, which is normocaloric, has been used to treat epilepsy in children. Other forms of ketogenic diet differ in fat-to-protein and carbohydrate ratios, and these include modified Atkins diet, low glycemic index treatment, and medium-chain triglyceride diet [8]. KD has also been used as a weight loss strategy [9] and to reduce insulin resistance in both type 1 and type 2 diabetes [10,11]. In this context, KD can be adapted to be vegetarian by including plant proteins rather than animal proteins, giving diabetic or obese patients the flexibility to choose their preferred diets. However, other studies have reported the side effects of KD on renal function and lipid profiles due to its high content of protein and fat [11].

Interestingly, the therapeutic potential of KD to improve seizure activity has been linked to its effect on the gut microbiota and related metabolites, although KD has been reported to reduce gut microbial diversity [12,13]. The gut microbiota has been described as an endocrine organ. It has a major impact on host health and is associated with several diseases [14]. The gut microbiota is highly affected by dietary intake, which plays a major role in modulating host metabolism in addition to shaping the gut microbiome [15,16]. Gut bacteria play an important role in digestion in the gastrointestinal tract, and commensal bacteria are pivotal for the synthesis and absorption of nutrients and metabolites such as lipids, amino acids, bile acids, vitamins, and short-chain fatty acids (SCFAs). SCFAs result from the fermentation of dietary fiber and resistant starch by the gut bacteria [17,18]. Furthermore, studies have reported the crucial role of the gut microbiota in modulating the homeostasis and function of innate and adaptive immune cells [19]. It is important to mention that the gut microbiota functions differently in different individuals due to the unique bacterial combination in each individual, which is related to inter-and intra-individual variation in humans [18]. Studies in humans [20] and animals [21] have shown that KD is able to alter the function of the gut microbiota in health [16]. Ang et al. reported that KD-associated gut microbiota is able to reduce the accumulation of Th17 cells, indicating an anti-inflammatory role of KD [16]. In addition, Kong et al. reported that fecal microbial transplantation from donors with KD was able to alleviate colitis in DSS-treated mice recipients [12]. The authors suggested that their results showed that the anti-inflammatory effects of KD may open the door to a therapeutic approach for IBD patients [12]. Moreover, an animal study by Olson et al. found that specific KD-associated bacteria were responsible for the anti-seizure effects. This effect was achieved by the KD-associated microbiota being able to modulate amino acid γ-glutamylation and hippocampal GABA/glutamate [21]. Furthermore, KD could lead to genetic variation within the gut microbiome, and the microbiome diversity could be altered by increasing the ratio of *Bacteroidetes* to *Firmicutes* [22]. However, more studies are still needed for long-term (>2 years), medium-term (>6 months –2 years), and short-term (<3 months) KD studies are essential to investigate its effect on the microbiome and determine whether it is directly related to the resulting weight loss or not [8].

Most studies on KD have used 16S rRNA gene sequencing or metagenome sequencing to assess the relative abundance of bacteria, and the results from different studies, most often based on the 16S rRNA gene and metagenome sequencing, have been inconsistent. This difference is due to the fact that metagenomics is able to detect less abundant taxa, and sometimes, these less abundant genera could be more biologically meaningful than the highly abundant ones that are only detected by 16S rRNA sequencing [23]. For characterizing microbial changes and showing bacteria and host interactions, high-throughput sequencing techniques have emerged to be the most important. Nevertheless, data interpretation from these techniques is based on relative abundance, and it disregards the absolute abundance or total bacterial load. In specific cases, such as addressing biological problems like community interactions, absolute abundance is necessary. As such, absolute abundance is considered more important than relative abundance, and the interpretation of microbiota data based on only relative abundance can be ambiguous. The different approaches to absolute quantification are diverse. For instance, the absolute quantification of specific taxa can be calculated by multiplying the relative abundance of the taxa generated by the 16S rRNA amplicon sequencing by the total cell count [24]. However, bias could potentially occur when using different absolute and relative quantification tools, such as 16S rRNA copy number discrepancy and qPCR primer specificity [24,25]. Another PCR-independent method that can directly enumerate specific taxa is fluorescence in situ hybridization (FISH), which uses a fluorescent probe to hybridize complementary sequences in the target cells. Due to its high sensitivity, FISH is able to detect and quantify low-abundance microbes [24]. It can be used to calculate relative taxon abundance, and in an optimized methodological approach like flow cytometry or microscopy, it can even estimate absolute taxon abundance [24,25,26]. The aim of the present study was to determine the differences in gut microbiota between healthy C57/BL6J mice fed a Western diet (WD), and those fed a KD, using fluorescence in situ hybridization (FISH) to study the absolute and relative abundance of different bacterial taxa.

## 2. Materials and Methods

### 2.1. Mice and Dietary Intervention

C57BL/6J mice (N = 20, *n* = 10/group) were maintained under standard conditions in a specific pathogen-free environment at 12 h day-night cycles according to the FELASA recommendations with food and water *ad libitum*. Following a one-week acclimatization period, female mice were divided randomly into homogeneous groups according to their weight and age (10–12 weeks of age) and fed an *ad libitum* Western or ketogenic diet for four weeks. The Western diet contained 18% protein, 59% carbohydrates, and 23% fat in terms of energy content. The ketogenic diet contained 15% protein, 1.4% carbohydrates, and 83.6% fat. Health status, such as the observation of behavior, physical assessment, and body weight, was monitored twice per week throughout the intervention. At the end of the dietary intervention, fecal samples from individual mice were fixed in modified Carnoy’s solution (ethanol:glacial acetic acid: chloroform, 6:6:1, *v:v:v*) for at least 24 h at room temperature [27]. Fecal samples were embedded in paraffin, and a 4 µm thick section was cut from each sample for fluorescence in-situ hybridization (FISH). To evaluate the ketotic state of diet-fed mice, blood concentrations of β-hydroxybutyrate, as an indicator of ketone body levels as well as blood glucose levels, were measured at the end of the experiment using a ketone and glucose meter.

The experiments were approved by the State Animal Care Committee (Landesamt für Gesundheit und Soziales, Berlin, Germany; approval code G0047/15) and performed according to the guidelines for the care and use of laboratory animals adopted by the U.S. National Institutes of Health, and the ARRIVE guidelines.

### 2.2. Fluorescence In-Situ Hybridization (FISH)

The Fecal microbiota was investigated using FISH analysis with ribosomal RNA-derived probes. Hybridization was performed on 4 µM thick sections of Carnoy-fixed and paraffin-embedded stool samples [28]. The samples were collected after 4 weeks of feeding.

The bacteria were quantified using group-specific C3 probes. The FITC-marked universal probe was used in each hybridization to evaluate the number of all bacteria, and C5 marked probes with a different specificity to C3 probes were used to determine the spatial relationship between different bacterial groups. Only signals that hybridized with a specific FISH probe and the universal FISH probe that did not hybridize with specific FISH probes from unrelated bacterial groups were evaluated [29].

Bacterial concentrations in homogeneous populations were enumerated visually in one of the 10 × 10 fields of the ocular raster, corresponding to 10 µm × 10 µm of the section surface at a magnification of ×1000. This number was assigned to a concentration of ×10^9^ bacteria/mL, which was equivalent to the formula used previously [28].

In the case of uneven distribution of bacteria over the microscopic field, the positive signals were enumerated in 10 fields of the ocular raster along the gradient of distribution, and an average was used after dividing by 10.

### 2.3. Investigated Bacterial Groups and FISH Probes

A total of 48 bacterial FISH probes were applied. Seven of these probes (ACI623, Bcv13b, CAP365, EUB338 II, Pce, Phasco741, Veil223) were excluded from the analysis because the bacteria detected by these probes were not specific to the mouse intestine. The names of the FISH probes are listed according to abbreviations of the probeBase online resource (https://probebase.csb.univie.ac.at (accessed on 16 February 2022)) [30]. Details of the FISH probe specificity and hybridization conditions are provided in probeBase.

The probes in Table 1 were ordered alphabetically into subgroups according to their abundance and specificity, as described in the Results section.

### 2.4. Statistical Analysis

Statistical analyses were performed using the GraphPad Prism 8.1.2 software (San Diego, CA, USA). Since some data were not normally distributed, statistical significance was determined using the non-parametric Mann-Whitney U test. Differences were considered statistically significant at *p* < 0.05. The data are expressed as individual values ± SEM.

## 3. Results

### 3.1. Efficacy of the Ketogenic Diet in Mice

**Figure 1:**
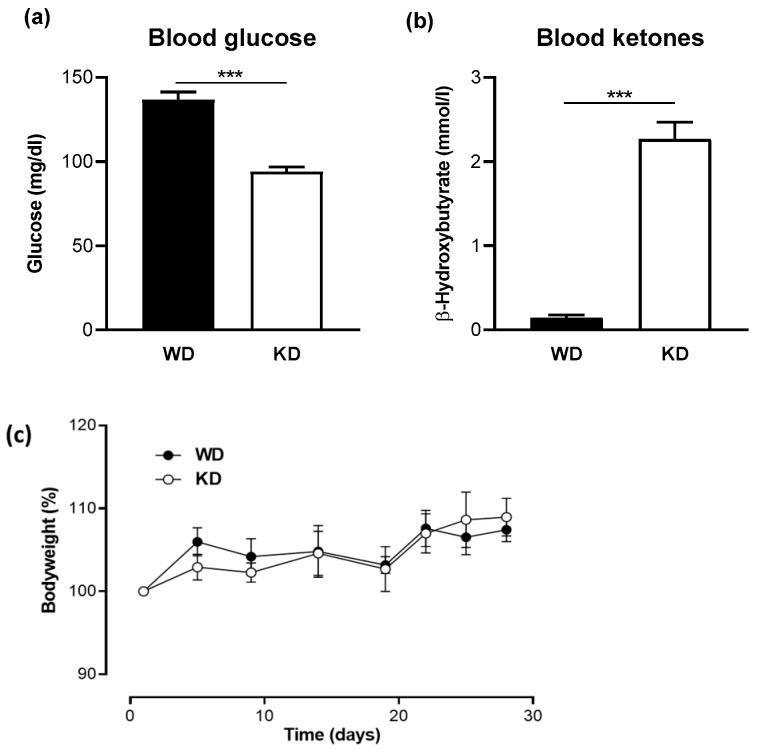
A Ketogenic diet leads to low glucose and high ketone levels in the blood. (**a**) Blood glucose levels and (**b**) blood β-hydroxybutyrate concentrations in mice fed a western diet (WD) or a ketogenic diet (KD) for four weeks (*n* = 10/group). Data are represented as mean ± SEM. Statistical significance was assessed using the Mann-Whitney U test (*** *p* < 0.001). (**c**) No significant change in body weight was observed between the WD- and KD-fed mice.

Mice were fed for 4 weeks with a WD in the control group and with KD in the treatment group. As expected, a significant reduction in blood glucose concentration and a significant increase in ketone levels were observed in the KD-fed group compared with those in the WD-fed group (Figure 1a,b). However, no significant change in body weight was observed in either group (Figure 1c).

### 3.2. Eligibile FISH Probes Used for Analysis of the Stool Microbiome

Depending on their prevalence and abundance, the investigated bacterial groups were divided into two categories: “substantial” and “marginal.” **Substantial bacterial groups** were found in at least 20% of the fecal samples at concentrations higher than 10^9^ bacteria/mL. **Marginal bacterial groups** showed low occurrence as well as marginal concentrations. They occurred in less than 20% of fecal samples at concentrations below 10^9^ bacteria/mL and, in most cases, lower than 10^8^ bacteria/mL. According to this classification, 19 of the FISH probes represented substantial bacterial groups, and 22 of the probes presented marginal bacterial groups (Table 1). Because the marginal bacterial groups did not significantly contribute to the overall colonic biomass, they were excluded from the subsequent evaluation. This specific definition and division of bacterial groups, depending on their prevalence, were previously introduced and described by Swidsinski et al. [31].

The bacteria were subdivided into highly conserved and individual bacterial groups. Three bacteria detected using CF319a (most *Flavobacteria*, some *Bacteroidetes)*, CFB560 (subgroup of *Bacteroidetes*, *CFB* division), and MIB661 (mouse intestinal bacteria) probes were consistently present in the fecal samples of mice fed the WD and KD at concentrations between 10 × 10^9^ and 25 × 10^9^ bacteria/mL, contributing to approximately half of the colonic microbiota in each mouse. The invariability and predominance of these three bacterial groups in C57BL/6J mice contributed to a major part of the microbiome and were designated as **highly conserved bacteria**. All other substantial bacterial groups were present only in a subset of the mice and were designated as **individual bacteria**. As their concentrations reached 10^10^ bacteria/mL, they contributed substantially to the fecal biomass, comprising approximately 50% of the biomass.

### 3.3. Effect of the Ketogenic Diet on the Fecal Microbiome in Mice

The fecal microbiota of WD- and KD-fed C57BL/6J mice were analyzed by FISH using 41 previously described probes (Table 1).

**Figure 2:**
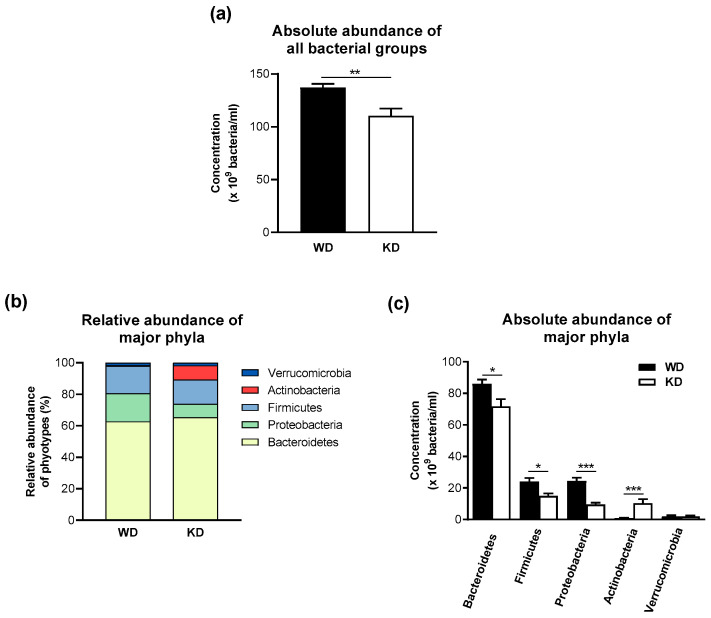
The ketogenic diet-induced moderate compositional changes and lower numbers of bacteria in the fecal microbiota of wild-type mice. (**a**) Total bacterial number, (**b**) relative abundance of major phyla, and (**c**) absolute microbiota composition at the phylum level. Data are represented as mean ± SEM (*n* = 10/group). Statistical significance was assessed using the Mann-Whitney U test (* *p* < 0.05, ** *p* < 0.01, *** *p* < 0.001).

Feeding mice with the KD decreased the overall number of bacteria (Figure 2a). This observation was also reflected by the reduction in the total number of highly conserved bacterial groups (Table 2). The total numbers of the marginal bacterial group and all individual bacteria were not changed by the KD (Table 2). The decrease in the highly conserved bacterial groups in the stool samples of KD-fed mice was mainly due to a significant reduction in bacteria detected by the MIB661 probe, specific for several *Bacteroides* species. Bacteria detected by the CF319a and CFB560 probes had similarly high concentrations in mice fed the KD and WD.

Although the total number of individual bacterial subgroups did not change in the KD-fed group, the most distinct changes in single bacteria were found in this group, with statistically significant differences found for 7 of the 16 investigated individual bacterial groups (Table 2). The most abundant individual bacterial groups in WD-fed mice were *Burkholderia cepacia* (Burcep probe), followed by the *Eubacterium hallii* group (Ehal1469 probe), and *Bacteroides putredinis* (Bputre698 probe), and the numbers of these three bacterial groups were lower in KD-fed mice (Table 2). In addition, the number of *Sphingomonas*/*Erythrobacter* (SPH492 probe) was lower in the KD group (Table 2). The dominant individual bacterial groups in the KD-fed mice were *Bacteroides distasonis* (Bdis656 probe), *Eubacterium cylindroides* (Ecyl387 probe), and *Bacteroides spp*. (Bac303 probe) (Table 2). Three of the investigated individual bacterial groups showed an increase in KD-fed as compared to WD-fed mice by approximately 200 to 4600%: *Atopobium* cluster (Ato291 probe), *Bifidobacterium* spp. (Bif662 probe), and the *Eubacterium cylindroides* group (Ecyl387 probe) (Table 2).

As mentioned before, differences in single marginal bacterial groups were not statistically evaluated between WD- and KD-fed mice.

The three most abundant phyla in the stool of WD-fed mice were *Bacteroidetes* (62.8%), *Proteobacteria* (17.8%), and *Firmicutes* (17.3%), whereas *Verrucomicrobia* (1.5%) and *Actinobacteria* (0.6%) were only present in a minor proportion in the stool samples of mice consuming the WD (Figure 2b). In contrast, the KD-fed group showed a higher relative abundance of *Actinobacteria* (9.1%) and a lower abundance of *Proteobacteria* (8.5%) (Figure 2b). These compositional differences were confirmed at the level of absolute numbers, as KD-fed mice showed a higher absolute abundance of *Actinobacteria* and a lower absolute abundance of the phylum *Proteobacteria* (Figure 2c). Interestingly, the absolute abundances of *Bacteroidetes* and *Firmicutes* were lower in the KD-fed group in comparison to the WD-fed group, and the average ratio of *Firmicutes*/*Bacteroidetes* was not significantly different between the WD-fed (0.280 ± 0.084) and the KD-fed groups (0.220 ± 0.091).

## 4. Discussion

Diet plays a major role in causing 50% of gut microbial variations in mice and 20% in humans, making it a potential strategy for disease management via modulation of the gut microbiota [32,33,34]. Studies reporting the effects of KD on the gut microbiome of both humans and mice have shown that KD leads to a lower overall alpha diversity [8,35]. This effect leads to lower SCFAs production, which mainly results from the fermentation of dietary fibers and nondigestible carbohydrates by the gut microbiota [8,35]. Studies have demonstrated through high-throughput DNA sequencing technologies in large-scale 16S rRNA and shotgun metagenomics different changes in the composition of the colonic microbiome upon KD in both humans and mice [16,21,35,36,37,38,39]. These studies reported the influence of the KD on bacterial taxa, richness, and diversity. They mentioned the positive effect of the KD in reshaping the gut microbiota and its biological functions and its negative effects like decreased diversity and increased number of pro-inflammatory bacteria [11]. In addition, using the FISH technique, Swidsinski et al. showed that there was a decrease in the diversity and total concentration of bacteria in the gut of humans consuming KD [31], which was also observed in wild-type C57Bl/6J mice in our study. However, because most studies are carried out in the short term, they are disease-associated, which limits their generalizability to the overall population.

In our study, we also used the FISH technique, but we could not have a final conclusion on the whole biodiversity or composition of the microbiota, as FISH only detects bacteria at concentrations higher than 10^5^ per ml. Nevertheless, Swidsinski et al. explained that even though the information provided by sequence analysis on the physical abundance and contribution of bacteria to bio-fermentation is substandard, their abundance within the fecal matter directly expresses their bio-fermenting power [40].

Our data quantifying bacterial participants demonstrated a clear difference in bacterial concentration and composition after KD compared with WD in C57BL/6J mice. For instance, the total number of bacteria in the fecal samples of mice consuming KD was reduced compared to those consuming WD, confirming previous data [41]. Feeding mice with the KD resulted in higher levels of bacteria belonging to the phylum *Actinobacteria.* Numerous reports have shown that members of *Actinobacteria*, such as *Bifidobacterium* spp., play a protective role against colitis and are able to reduce the overall level of systemic inflammation [42,43,44,45]. In addition, the significant decrease in blood glucose levels observed after 4 weeks of KD was consistent with the significant increase in *Actinobacteria*, and in particular *Bifidobacterium* spp., which have previously been reported to have lower blood glucose and act as an anti-diabetic agent [46]. This significant increase in the *Bifidobacterium* spp. after KD may indicate that KD can have a possible beneficial effect on the inflammatory status, as well as on glucose homeostasis.

On the other hand, in our study, KD in mice led to a decrease in the number of *Proteobacteria*. Members of the *Proteobacteria* phylotype have been shown to play a role in the onset and progression of ulcerative colitis and inflammatory diseases, such as Crohn’s disease [45,47,48,49,50]. *Proteobacteria* represent a minor part of the healthy gut microbiota, but their disproportionate increase leads to inflammation [51]; as such, *Proteobacteria* have been considered as a microbial signature of intestinal dysbiosis [52]. Similar to our results, Kong et al. showed that KD was able to reduce the abundance of *Proteobacteria* in mice with DSS-induced colitis [12]. This may be due to the low intake of carbohydrates and sugars in the KD, which can be fermented by *Proteobacteria,* leading to their increase [51]. However, in their systemic review, Kaviyarasan et al. found several studies in which KD was associated with an increase in the number of *Proteobacteria* [53].

Interestingly, we found no differences in the abundance of *Verrucomicrobia* between WD- and KD-fed mice. This is an important finding as this taxon contains members such as *Akkermansia muciniphila*, whose presence has been associated with gut health and reversal of experimental colitis [54,55]. Our results were not consistent with those of Ma et al., and Olson et al., who showed an increase in *A. muciniphila* after a KD in mice [8,21,36]. *A. muciniphila* has been reported to have anti-diabetic effects, leading to an improvement in insulin secretion and glucose homeostasis [56]. This was confirmed in the studies by Ma et al. and Olson et al., where a decrease in glucose levels was associated with an increase in *A. muciniphila* numbers after KD [8,21,36]. However, another study by Newell et al. found a significant decrease in *A. muciniphila* numbers in BTBR mice following a KD compared to a chow diet [41], possibly due to the very low carbohydrate content of the KD, which serves as a fuel source for microorganisms such as *A. muciniphila* [57]. This effect was only observed in BTBR mice and not wild-type mice, suggesting a disease-related mechanism [41]. Looking at the different results observed in C57BL/6 mice, it is noteworthy that *A. muciniphila* was significantly increased after a 16-week ketogenic diet [36], but not after 4 weeks (our study) or 14 days [41], suggesting that a longer duration of KD may be required to cause a significant increase in *A. muciniphila.*

Looking more closely at the individual bacteria detected by the FISH probes, we found a significant increase in the *Atopobium* cluster in the KD group compared to the WD group, which has been implicated in the pathogenesis of several diseases such as type 2 diabetes mellitus [58], and ulcerative colitis [59]. However, we also observed a significant decrease in the concentration of opportunistic pathogens associated with infections, such as *Sphingomonas* and *Erythrobacter,* in the KD group. 

Furthermore, our results showed that *Bacteroidetes* were overrepresented and *Firmicutes* were underrepresented in comparison to previous studies based on 16S rRNA sequencing [37] or metagenome sequencing. This may be due to the low number of probes specifically designed for the detection of *Firmicutes* in the murine gut. Therefore, claims based on the *Firmicutes/Bacteroides* ratio should be carefully considered. Here, the 4-week KD in C57Bl/6J mice did not have a significant effect on the *Firmicutes* to *Bacteroidetes* ratio compared to the WD. As *Firmicutes* and *Bacteroidetes* represent more than 90% of the gut microbial community, the ratio of *Firmicutes* to *Bacteroidetes* is considered important for microbial balance and gut health [11]. This composition is relatively unaffected by acute perturbations but is affected by continuous exposure to various stress factors that can have an impact on host health [60].

Evidence from different studies has shown a clear effect of diet on the gut microbiome and a clear relationship between the state of the gut microbiome and chronic diseases such as type 2 diabetes mellitus (T2DM) and cardiovascular disease (CVD) [61]. For instance, the beneficial effects of the KD on the gut microbiota have been implicated in metabolic health [8]. Defeudis et al. stated that a very low carbohydrate ketogenic diet (VLCKD) might represent an effective strategy for treating T2DM and obesity as it results in ketosis due to low carbohydrate content, and this effect has been related to the impact of VLCKD on the gut microbiota [62]. In addition, ketosis has been associated with different biochemical and physiological mechanisms that exert a systemic anti-inflammatory effect, which in turn has a direct effect on cardiovascular diseases [63]. Furthermore, VLCKD is reported to improve the diversity of the microbiota by counteracting *Proteobacteria* leading to weight loss and favouring *Firmicutes, Ruminococcaceae*, and *Mogibacteriaceae* [62]. In our study, we were able to confirm a decrease in *Proteobacteria* in the KD-fed mice. However, we did not observe a corresponding increase in the other genera, which could be due to the short-term period of the diet followed in our study. Furthermore, the proven anti-inflammatory effects of KD reported in different studies make it a potential strategy for the prevention or treatment of CVD. Another anti-inflammatory factor in KD is the elimination of pro-inflammatory simple sugars, which has been reflected in CVD improvement because the restriction of carbohydrate content has shown anti-inflammatory benefits in the case of cardiometabolic health. As such, these studies recommend a high-fat, well-composed KD that is rich in omega-3 polyunsaturated fatty acids due to its anti-inflammatory and cardioprotective effects [63].

In conclusion, KD led to a lower total number of bacteria in the guts of mice. A limitation to consider is the duration of the dietary intervention, as longer durations have been shown to have a stronger effect on shaping the gut microbiota. Our study was also limited by the fact that the fecal microbiota analysis was performed using the FISH technique. This technique does not allow an in-depth study of all the major species involved in health and disease; therefore, we still lack data to make a definitive conclusion about the effect of KD at the species level. Another limitation of the study is that the effect of KD on the fecal microbiota was only investigated in female mice, and the estrous cycle was not determined, as recent studies have shown sex differences in response to KD [64,65]. Therefore, future studies are needed to evaluate possible sex-related effects on the gut microbiota associated with KD. However, the preliminary data we have obtained shows that the KD can alter the gut microbiota composition in female wild-type mice, which could potentially have anti-inflammatory and anti-diabetic effects. Accordingly, the gut microbiota may be considered a target for the prevention or treatment of diseases associated with a western diet and lifestyle. However, more preclinical and clinical studies should be conducted to further investigate the effect of the KD on different diseases and to reach a definitive conclusion, as the results are still controversial among different studies.

## Figures and Tables

**Table 1:** Applied FISH probes.

Marginal Groups (*N* = 22)	Substantial Groups (*N* = 19)
Bcat187 (*Bifidobacterium catenulatum* group)	***Highly-conserved bacteria*** (*N = 3*)
Bif1278 (*Bifidobacterium* spp.)	CF319a (most *Flavobacteria*, some *Bacteroidetes*)
Bif153 (Genus *Bifidobacterium*)	CFB560 (subgroup of *Bacteroidetes*, *CFB* division)
Bifado182 (*Bifidobacterium adolescentis*)	MIB661 (mouse intestinal bacteria)
Bifado434 (*Bifidobacterium adolescentis)*	***Individual*** (*N = 16*)
Blon1004 (*Bifidobacterium longum*)	Ato291 (*Atopobium* cluster)
Ceut705 (*Coprococcus eutactus*, *Coprococcus sp.*)	Bac303 (most *Bacteroidaceae*)
Chis150 (*Clostridium histolyticum* group)	Bdis656 (*Bacteroides distasonis*)
Clit135 (*Clostridium lituseburense* group including *Clostridium difficile*)	Bif662 (*Bifidobacterium* spp.)
Cor653 (*Coriobacterium* group)	Bputre698 (*Bacteroitedes putredinis*)
CST440 (Group 1 clones closely related to *Clostridium stercorarium*)	Burcep (*Burkholderia cepacia*)
Ebac1790 (*Enterobacteriaceae*)	Ecyl387 (*Eubacterium cylindroides* group)
Efaec (*Enterococcus faecalis*, *Enterococcus sulfuricus*)	Ehal1469 (*Eubacterium hallii* group)
Erec482 (*Eubacterium rectale*-*Clostridium coccoides* group)	Eram997 (*Eubacterium ramulus*)
Fprau0645 (*Faecalibacterium prausnitzii*)	EubIII Phylum (*Verrucomicrobia*)
Lab158 (*Lactobacillus sp*., *Enterococcus sp.*)	ProCo1264 (*Ruminococcus productos*)
Pnig657 (*Prevotella nigrescens)*	Rfla729 (*Ruminococcus albus*, *Ruminococcus flavefaciens*)
Rbro730 (*Clostridium sporosphaeroides*, *Ruminococcus bromii*, *Clostridium leptum*)	SFB1 (Segmented filamentous bacteria)
Strc493 (*most Streptococcus* spp.)	SPH492 (*Sphingomonas, Erythrobacter*)
Urobe63a (*Ruminococcus obeum*-like)	SUBU1237 (*Burkholderia* spp., *Sutterella* spp.)
Urobe63b *(Ruminococcus obeum*-like)	Ver620 (*Verrucomicrobium*)
Y (*Yersinia)*	

**Table 2:** Mean microbial concentrations ± SD (×10^9^ bacteria/mL) of all substantial bacterial groups in stool samples of mice fed a Western diet (WD) and a ketogenic diet (KD). The increase (↑) or decrease (↓) in bacterial % between the two groups is indicated.

	WD	KD	Change (%)	*p* Value
Bacterial composition as % of bacteria positivein each mouse	82.93 ± 5.86	69.27 ± 4.02	↓ 17%	<0.001
All bacteria	137.26 ± 10.80	110.57 ± 21.09	↓ 19%	0.0039
All substantial bacteria	135.99 ± 10.92	107.08 ± 18.63	↓ 21%	0.0021
All marginal bacteria	1.27 ± 0.69	3.49 ± 5.79		ns
**HIGHLY-CONSERVED**				
All highly-conserved bacteria	63.20 ± 5.35	43.90 ± 9.23	↓ 30%	<0.001
CF319a (most *Flavobacteria*, some *Bacteroidetes)*	22.90 ± 5.40	20.50 ± 7.88		ns
CFB560 (subgroup of *Bacteroidetes*, *CFB* division)	15.70 ± 6.09	11.00 ± 4.40		ns
MIB661 (mouse intestinal bacteria)	24.60 ± 3.81	12.40 ± 3.81	↓ 50%	<0.001
**INDIVIDUAL**				
All individual bacteria	72.79 ± 13.52	63.18 ± 11.82		ns
Ato291 (*Atopobium* cluster)	0.07 ± 0.06	3.31 ± 2.81	↑ 4629%	<0.001
Bac303 (most *Bacteroidaceae)*	6.50 ± 4.25	9.40 ± 5.04		ns
Bdis656 (*Bacteroides distasonis)*	7.35 ± 3.67	12.40 ± 6.04		ns
Bif662 (*Bifidobacterium* spp.)	0.35 ± 0.41	3.98 ± 2.77	↑ 1037%	<0.001
Bputre698 (*Bacteroides putredinis)*	8.90 ± 2.69	6.00 ± 1.89	↓ 33%	0.0138
Burcep (*Burkholderia cepacia)*	13.20 ± 6.91	2.40 ± 3.24	↓ 82%	0.0019
Ecyl387 (*Eubacterium cylindroides* group)	3.25 ± 3.28	10.90 ± 6.56	↑ 235%	0.0044
Ehal1469 (*Eubacterium hallii* group)	10.78 ± 6.48	0.30 ± 0.44	↓ 97%	<0.001
Eram997 (*Eubacterium ramulus)*	2.79 ± 3.55	0.28 ± 0.34		ns
EubIII Phylum (*Verrucomicrobia)*	0.96 ± 0.92	1.01 ± 1.04		ns
ProCo1264 (*Ruminococcus productos)*	2.90 ± 1.73	1.84 ± 0.69		ns
Rfla729 (*Ruminococcus albus, Ruminococcus flavefaciens)*	1.15 ± 1.24	0.15 ± 0.35		ns
SFB1 (Segmented filamentous bacteria)	3.79 ± 2.57	3.40 ± 2.95		ns
SPH492 (*Sphingomonas, Erythro-bacter)*	2.47 ± 3.04	0.03 ± 0.03	↓ 99%	<0.001
SUBU1237 (*Burkholderia* spp., *Sutterella* spp.)	8.70 ± 4.52	7.00 ± 2.54		ns
Ver620 (*Verrucomicrobium)*	1.24 ± 1.98	0.95 ± 1.42		ns

## Data Availability

Not applicable.

## References

[1] Zhu H., Bi D., Zhang Y., Kong C., Du J., Wu X., Wei Q., Qin H. (2022). Ketogenic diet for human diseases: The underlying mechanisms and potential for clinical implementations. Signal Transduct. Target. Ther..

[2] Wijnen B.F.M., de Kinderen R.J.A., Lambrechts D., Postulart D., Aldenkamp A.P., Majoie M., Evers S. (2017). Long-term clinical outcomes and economic evaluation of the ketogenic diet versus care as usual in children and adolescents with intractable epilepsy. Epilepsy Res..

[3] Gano L.B., Patel M., Rho J.M. (2014). Ketogenic diets, mitochondria, and neurological diseases. J. Lipid Res..

[4] Boison D. (2017). New insights into the mechanisms of the ketogenic diet. Curr. Opin. Neurol..

[5] Srivastava S., Pawar V.A., Tyagi A., Sharma K.P., Kumar V., Shukla S.K. (2023). Immune Modulatory Effects of Ketogenic Diet in Different Disease Conditions. Immuno.

[6] Milder J., Patel M. (2012). Modulation of oxidative stress and mitochondrial function by the ketogenic diet. Epilepsy Res..

[7] Puchalska P., Crawford P.A. (2017). Multi-dimensional Roles of Ketone Bodies in Fuel Metabolism, Signaling, and Therapeutics. Cell Metab..

[8] Attaye I., van Oppenraaij S., Warmbrunn M.V., Nieuwdorp M. (2021). The Role of the Gut Microbiota on the Beneficial Effects of Ketogenic Diets. Nutrients.

[9] Dashti H.M., Mathew T.C., Khadada M., Al-Mousawi M., Talib H., Asfar S.K., Behbahani A.I., Al-Zaid N.S. (2007). Beneficial effects of ketogenic diet in obese diabetic subjects. Mol. Cell. Biochem..

[10] Bolla A.M., Caretto A., Laurenzi A., Scavini M., Piemonti L. (2019). Low-Carb and Ketogenic Diets in Type 1 and Type 2 Diabetes. Nutrients.

[11] Paoli A., Mancin L., Bianco A., Thomas E., Mota J.F., Piccini F. (2019). Ketogenic Diet and Microbiota: Friends or Enemies?. Genes.

[12] Kong C., Yan X., Liu Y., Huang L., Zhu Y., He J., Gao R., Kalady M.F., Goel A., Qin H. (2021). Ketogenic diet alleviates colitis by reduction of colonic group 3 innate lymphoid cells through altering gut microbiome. Signal Transduct. Target. Ther..

[13] Rawat K., Singh N., Kumari P., Saha L. (2021). A review on preventive role of ketogenic diet (KD) in CNS disorders from the gut microbiota perspective. Rev. Neurosci..

[14] Brown J.M., Hazen S.L. (2015). The gut microbial endocrine organ: Bacterially derived signals driving cardiometabolic diseases. Annu. Rev. Med..

[15] Cani P.D., Van Hul M., Lefort C., Depommier C., Rastelli M., Everard A. (2019). Microbial regulation of organismal energy homeostasis. Nat. Metab..

[16] Ang Q.Y., Alexander M., Newman J.C., Tian Y., Cai J., Upadhyay V., Turnbaugh J.A., Verdin E., Hall K.D., Leibel R.L. (2020). Ketogenic Diets Alter the Gut Microbiome Resulting in Decreased Intestinal Th17 Cells. Cell.

[17] Portincasa P., Bonfrate L., Vacca M., De Angelis M., Farella I., Lanza E., Khalil M., Wang D.Q., Sperandio M., Di Ciaula A. (2022). Gut Microbiota and Short Chain Fatty Acids: Implications in Glucose Homeostasis. Int. J. Mol. Sci..

[18] Rinninella E., Raoul P., Cintoni M., Franceschi F., Miggiano G.A.D., Gasbarrini A., Mele M.C. (2019). What is the Healthy Gut Microbiota Composition? A Changing Ecosystem across Age, Environment, Diet, and Diseases. Microorganisms.

[19] Brestoff J.R., Artis D. (2013). Commensal bacteria at the interface of host metabolism and the immune system. Nat. Immunol..

[20] Mardinoglu A., Wu H., Bjornson E., Zhang C., Hakkarainen A., Räsänen S.M., Lee S., Mancina R.M., Bergentall M., Pietiläinen K.H. (2018). An Integrated Understanding of the Rapid Metabolic Benefits of a Carbohydrate-Restricted Diet on Hepatic Steatosis in Humans. Cell Metab..

[21] Olson C.A., Vuong H.E., Yano J.M., Liang Q.Y., Nusbaum D.J., Hsiao E.Y. (2018). The Gut Microbiota Mediates the Anti-Seizure Effects of the Ketogenic Diet. Cell.

[22] Dowis K., Banga S. (2021). The Potential Health Benefits of the Ketogenic Diet: A Narrative Review. Nutrients.

[23] Durazzi F., Sala C., Castellani G., Manfreda G., Remondini D., De Cesare A. (2021). Comparison between 16S rRNA and shotgun sequencing data for the taxonomic characterization of the gut microbiota. Sci. Rep..

[24] Wang X., Howe S., Deng F., Zhao J. (2021). Current Applications of Absolute Bacterial Quantification in Microbiome Studies and Decision-Making Regarding Different Biological Questions. Microorganisms.

[25] Props R., Kerckhof F.M., Rubbens P., De Vrieze J., Hernandez Sanabria E., Waegeman W., Monsieurs P., Hammes F., Boon N. (2017). Absolute quantification of microbial taxon abundances. ISME J..

[26] Daims H., Ramsing N.B., Schleifer K.H., Wagner M. (2001). Cultivation-independent, semiautomatic determination of absolute bacterial cell numbers in environmental samples by fluorescence in situ hybridization. Appl. Environ. Microbiol..

[27] Dorffel Y., Swidsinski A., Loening-Baucke V., Wiedenmann B., Pavel M. (2012). Common biostructure of the colonic microbiota in neuroendocrine tumors and Crohn’s disease and the effect of therapy. Inflamm. Bowel Dis..

[28] Swidsinski A., Loening-Baucke V., Kirsch S., Doerffel Y. (2010). Functional biostructure of colonic microbiota (central fermenting area, germinal stock area and separating mucus layer) in healthy subjects and patients with diarrhea treated with Saccharomyces boulardii. Gastroenterol. Clin. Biol..

[29] Swidsinski A. (2006). Standards for bacterial identification by fluorescence In situ hybridization within eukaryotic tissue using ribosomal rRNA-based probes. Inflamm. Bowel Dis..

[30] Greuter D., Loy A., Horn M., Rattei T. (2016). probeBase—An online resource for rRNA-targeted oligonucleotide probes and primers: New features 2016. Nucleic Acids Res..

[31] Swidsinski A., Dorffel Y., Loening-Baucke V., Gille C., Goktas O., Reisshauer A., Neuhaus J., Weylandt K.H., Guschin A., Bock M. (2017). Reduced Mass and Diversity of the Colonic Microbiome in Patients with Multiple Sclerosis and Their Improvement with Ketogenic Diet. Front. Microbiol..

[32] Zhang C., Zhang M., Wang S., Han R., Cao Y., Hua W., Mao Y., Zhang X., Pang X., Wei C. (2010). Interactions between gut microbiota, host genetics and diet relevant to development of metabolic syndromes in mice. ISME J..

[33] David L.A., Materna A.C., Friedman J., Campos-Baptista M.I., Blackburn M.C., Perrotta A., Erdman S.E., Alm E.J. (2014). Host lifestyle affects human microbiota on daily timescales. Genome Biol..

[34] Leeming E.R., Johnson A.J., Spector T.D., Le Roy C.I. (2019). Effect of Diet on the Gut Microbiota: Rethinking Intervention Duration. Nutrients.

[35] Zhang Y., Zhou S., Zhou Y., Yu L., Zhang L., Wang Y. (2018). Altered gut microbiome composition in children with refractory epilepsy after ketogenic diet. Epilepsy Res..

[36] Ma D., Wang A.C., Parikh I., Green S.J., Hoffman J.D., Chlipala G., Murphy M.P., Sokola B.S., Bauer B., Hartz A.M.S. (2018). Ketogenic diet enhances neurovascular function with altered gut microbiome in young healthy mice. Sci. Rep..

[37] Murtaza N., Burke L.M., Vlahovich N., Charlesson B., O’ Neill H., Ross M.L., Campbell K.L., Krause L., Morrison M. (2019). The Effects of Dietary Pattern during Intensified Training on Stool Microbiota of Elite Race Walkers. Nutrients.

[38] Lindefeldt M., Eng A., Darban H., Bjerkner A., Zetterström C.K., Allander T., Andersson B., Borenstein E., Dahlin M., Prast-Nielsen S. (2019). The ketogenic diet influences taxonomic and functional composition of the gut microbiota in children with severe epilepsy. NPJ Biofilms Microbiomes.

[39] Xie G., Zhou Q., Qiu C.Z., Dai W.K., Wang H.P., Li Y.H., Liao J.X., Lu X.G., Lin S.F., Ye J.H. (2017). Ketogenic diet poses a significant effect on imbalanced gut microbiota in infants with refractory epilepsy. World J. Gastroenterol..

[40] Swidsinski A., Dorffel Y., Loening-Baucke V., Gille C., Reisshauer A., Goktas O., Kruger M., Neuhaus J., Schrodl W. (2017). Impact of humic acids on the colonic microbiome in healthy volunteers. World J. Gastroenterol..

[41] Newell C., Bomhof M.R., Reimer R.A., Hittel D.S., Rho J.M., Shearer J. (2016). Ketogenic diet modifies the gut microbiota in a murine model of autism spectrum disorder. Mol. Autism.

[42] Duranti S., Gaiani F., Mancabelli L., Milani C., Grandi A., Bolchi A., Santoni A., Lugli G.A., Ferrario C., Mangifesta M. (2016). Elucidating the gut microbiome of ulcerative colitis: Bifidobacteria as novel microbial biomarkers. FEMS Microbiol. Ecol..

[43] Hidalgo-Cantabrana C., Algieri F., Rodriguez-Nogales A., Vezza T., Martinez-Camblor P., Margolles A., Ruas-Madiedo P., Galvez J. (2016). Effect of a Ropy Exopolysaccharide-Producing Bifidobacterium animalis subsp. lactis Strain Orally Administered on DSS-Induced Colitis Mice Model. Front. Microbiol..

[44] Srutkova D., Schwarzer M., Hudcovic T., Zakostelska Z., Drab V., Spanova A., Rittich B., Kozakova H., Schabussova I. (2015). Bifidobacterium longum CCM 7952 Promotes Epithelial Barrier Function and Prevents Acute DSS-Induced Colitis in Strictly Strain-Specific Manner. PLoS ONE.

[45] Zhang D., Wei C., Yao J., Cai X., Wang L. (2015). Interleukin-10 gene-carrying bifidobacteria ameliorate murine ulcerative colitis by regulating regulatory T cell/T helper 17 cell pathway. Exp. Biol. Med..

[46] Le T.K., Hosaka T., Nguyen T.T., Kassu A., Dang T.O., Tran H.B., Pham T.P., Tran Q.B., Le T.H., Pham X.D. (2015). Bifidobacterium species lower serum glucose, increase expressions of insulin signaling proteins, and improve adipokine profile in diabetic mice. Biomed. Res..

[47] Atherly T., Mosher C., Wang C., Hostetter J., Proctor A., Brand M.W., Phillips G.J., Wannemuehler M., Jergens A.E. (2016). Helicobacter bilis Infection Alters Mucosal Bacteria and Modulates Colitis Development in Defined Microbiota Mice. Inflamm. Bowel Dis..

[48] Mirsepasi-Lauridsen H.C., Halkjaer S.I., Mortensen E.M., Lydolph M.C., Nordgaard-Lassen I., Krogfelt K.A., Petersen A.M. (2016). Extraintestinal pathogenic Escherichia coli are associated with intestinal inflammation in patients with ulcerative colitis. Sci. Rep..

[49] Carvalho F.A., Koren O., Goodrich J.K., Johansson M.E., Nalbantoglu I., Aitken J.D., Su Y., Chassaing B., Walters W.A., González A. (2012). Transient inability to manage proteobacteria promotes chronic gut inflammation in TLR5-deficient mice. Cell Host Microbe.

[50] Vester-Andersen M.K., Mirsepasi-Lauridsen H.C., Prosberg M.V., Mortensen C.O., Träger C., Skovsen K., Thorkilgaard T., Nøjgaard C., Vind I., Krogfelt K.A. (2019). Increased abundance of proteobacteria in aggressive Crohn’s disease seven years after diagnosis. Sci. Rep..

[51] Satokari R. (2020). High Intake of Sugar and the Balance between Pro- and Anti-Inflammatory Gut Bacteria. Nutrients.

[52] Rizzatti G., Lopetuso L.R., Gibiino G., Binda C., Gasbarrini A. (2017). Proteobacteria: A Common Factor in Human Diseases. Biomed. Res. Int..

[53] Kaviyarasan S., Chung Sia E.L., Retinasamy T., Arulsamy A., Shaikh M.F. (2022). Regulation of gut microbiome by ketogenic diet in neurodegenerative diseases: A molecular crosstalk. Front. Aging Neurosci..

[54] Kang C.S., Ban M., Choi E.J., Moon H.G., Jeon J.S., Kim D.K., Park S.K., Jeon S.G., Roh T.Y., Myung S.J. (2013). Extracellular vesicles derived from gut microbiota, especially Akkermansia muciniphila, protect the progression of dextran sulfate sodium-induced colitis. PLoS ONE.

[55] Zhang Z., Wu X., Cao S., Wang L., Wang D., Yang H., Feng Y., Wang S., Li L. (2016). Caffeic acid ameliorates colitis in association with increased Akkermansia population in the gut microbiota of mice. Oncotarget.

[56] Yan X., Liu X.Y., Zhang D., Zhang Y.D., Li Z.H., Liu X., Wu F., Chen G.Q. (2021). Construction of a sustainable 3-hydroxybutyrate-producing probiotic Escherichia coli for treatment of colitis. Cell. Mol. Immunol..

[57] Flint H.J., Scott K.P., Duncan S.H., Louis P., Forano E. (2012). Microbial degradation of complex carbohydrates in the gut. Gut Microbes.

[58] Das T., Jayasudha R., Chakravarthy S., Prashanthi G.S., Bhargava A., Tyagi M., Rani P.K., Pappuru R.R., Sharma S., Shivaji S. (2021). Alterations in the gut bacterial microbiome in people with type 2 diabetes mellitus and diabetic retinopathy. Sci. Rep..

[59] Swidsinski A., Loening-Baucke V., Vaneechoutte M., Doerffel Y. (2008). Active Crohn’s disease and ulcerative colitis can be specifically diagnosed and monitored based on the biostructure of the fecal flora. Inflamm. Bowel Dis..

[60] Magne F., Gotteland M., Gauthier L., Zazueta A., Pesoa S., Navarrete P., Balamurugan R. (2020). The Firmicutes/Bacteroidetes Ratio: A Relevant Marker of Gut Dysbiosis in Obese Patients?. Nutrients.

[61] Sikalidis A.K., Maykish A. (2020). The Gut Microbiome and Type 2 Diabetes Mellitus: Discussing a Complex Relationship. Biomedicines.

[62] Defeudis G., Rossini M., Khazrai Y.M., Pipicelli A.M.V., Brucoli G., Veneziano M., Strollo F., Bellia A., Bitterman O., Lauro D. (2022). The gut microbiome as possible mediator of the beneficial effects of very low calorie ketogenic diet on type 2 diabetes and obesity: A narrative review. Eat. Weight Disord..

[63] Dyńka D., Kowalcze K., Charuta A., Paziewska A. (2023). The Ketogenic Diet and Cardiovascular Diseases. Nutrients.

[64] Cochran J., Taufalele P.V., Lin K.D., Zhang Y., Dale Abel E. (2018). Sex Differences in the Response of C57BL/6 Mice to Ketogenic Diets. Diabetes.

[65] Pontifex M.G., Vauzour D., Muller M. (2023). Sexual dimorphism in the context of nutrition and health. Proceedings of the Nutrition Society.

